# Using Complexity Assessment to Inform the Development and Deployment of a Digital Dashboard for Schizophrenia Care: Case Study

**DOI:** 10.2196/15521

**Published:** 2020-04-23

**Authors:** Andreas Gremyr, Boel Andersson Gäre, Trisha Greenhalgh, Ulf Malm, Johan Thor, Ann-Christine Andersson

**Affiliations:** 1 Department of Schizophrenia Spectrum Disorders (Psykiatri Psykos) Sahlgrenska University Hospital Mölndal Sweden; 2 Jönköping Academy for Improvement of Health and Welfare School of Health and Welfare Jönköping University Jönköping Sweden; 3 Futurum Academy for Health and Care Region Jönköping County Jönköping Sweden; 4 Nuffield Department of Primary Care Health Sciences University of Oxford Oxford United Kingdom; 5 Institute of Neuroscience and Physiology Sahlgrenska Academy University of Gothenburg Gothenburg Sweden

**Keywords:** health care, complexity, schizophrenia, coproduction, learning health systems

## Abstract

**Background:**

Health care is becoming more complex. For an increasing number of individuals, interacting with health care means addressing more than just one illness or disorder, engaging in more than one treatment, and interacting with more than one care provider. Individuals with severe mental illnesses such as schizophrenia are disproportionately affected by this complexity. Characteristic symptoms can make it harder to establish and maintain relationships. Treatment failure is common even where there is access to effective treatments, increasing suicide risk. Knowledge of complex adaptive systems has been increasingly recognized as useful in understanding and developing health care. A complex adaptive system is a collection of interconnected agents with the freedom to act based on their own internalized rules, affecting each other. In a complex health care system, relevant feedback is crucial in enabling continuous learning and improvement on all levels. New technology has potential, but the failure rate of technology projects in health care is high, arguably due to complexity. The Nonadoption, Abandonment, and challenges to Scale-up, Spread, and Sustainability (NASSS) framework and complexity assessment tool (NASSS-CAT) have been developed specifically to help identify and manage complexity in technology-related development projects in health care.

**Objective:**

This study aimed to use a pilot version of the NASSS-CAT instrument to inform the development and deployment of a point-of-care dashboard supporting schizophrenia care in west Sweden. Specifically, we report on the complexity profile of the project, stakeholders’ experiences with using NASSS-CAT, and practical implications.

**Methods:**

We used complexity assessment to structure data collection and feedback sessions with stakeholders, thereby informing an emergent approach to the development and deployment of the point-of-care dashboard. We also performed a thematic analysis, drawing on observations and documents related to stakeholders' use of the NASSS-CAT to describe their views on its usefulness.

**Results:**

Application of the NASSS framework revealed different types of complexity across multiple domains, including the condition, technology, value proposition, organizational tasks and pathways, and wider system. Stakeholders perceived the NASSS-CAT tool as useful in gaining perspective and new insights, covering areas that might otherwise have been neglected. Practical implications derived from feedback sessions with managers and developers are described.

**Conclusions:**

This case study shows how stakeholders can identify and plan to address complexities during the introduction of a technological solution. Our findings suggest that NASSS-CAT can bring participants a greater understanding of complexities in digitalization projects in general.

## Introduction

### Health Care Challenges

Health care is growing more complex and difficult to manage due to factors such as a rapidly expanding body of knowledge, a shift towards more people living with chronic disease and multi-morbidity [1], challenges in coordinating multiple providers and actors, and, not least, the need to include the preferences and values of the individuals seeking health care [2]. For an increasing number of individuals, interacting with health care means addressing more than just one illness or disorder, undergoing more than one treatment, and collaborating with more than one care provider [1].

### Schizophrenia as an Example

Addressing and adapting to complexity might be especially challenging in health care focusing on individuals with severe mental illnesses such as schizophrenia who are disproportionately affected by comorbid medical conditions [3]. With typical onset in early adulthood and a lifelong course, schizophrenia is among the top 10 disorders in terms of disability-adjusted life years lost [4]. Characteristic symptoms are hallucinations, delusions, and disturbances of thought. These features tend to make it challenging to establish and maintain relationships [5]. Treatment failure is common, increasing the risk of suicide [6], despite access to effective treatments [7]. Health and social services for persons with schizophrenia are marked by a high level of complexity. They involve a multimodal treatment approach with a range of treatments from a multiprofessional team, often requiring coordination with other providers (eg, primary care, care for other chronic disorders, social support, housing, and vocational rehabilitation) [5].

### Complex Adaptive Systems

Systems can be described either as simple (straight forward and predictable, with few components), complicated (predictable but with more interacting components), or complex (unpredictable and dynamic, where the whole is more than its constituent parts) [8]. Traditional linear cause-and-effect-thinking is not sufficient when studying systems that evolve in ways that are hard or even impossible to predict. Knowledge of complex adaptive systems can aid in understanding and studying health services [9]. A complex adaptive system is a collection of interconnected agents with the freedom to act based on their own internalized rules, affecting each other. These rules, in human-based complex adaptive systems, could be instinct and implicit mental models. Agents adapt in various ways through interactions, which causes the system to change over time [2]. Capability among individual agents in a complex system, that is “the extent to which an individual can adapt to change, generate new knowledge, and continue to improve their performance” [10], can be supported by minimum specifications (simple rules to guide behavior) and feedback loops, letting individuals gradually upgrade their internalized rules through experience. Relevant feedback on performance is crucial to enable continuous learning and improvement at all levels of a health care system, from the level of individual patients to organizational management and policy levels, potentially enabled by the use of new technologies [11-15].

### New Technology: A Blessing or a Curse?

The use of new technologies, argue Pavel et al [16], is essential to achieving personalized, evidence-based, and economically viable health care. Meanwhile, experiences so far reveal significant challenges. Uptake of “disruptive” technologies in health care is slow [17,18], and fundamental quality, safety, and cost problems have not been resolved by digitalization [19]. Moreover, the failure rate of technology projects in health care is high; large and complex projects often tend to fail to deliver anticipated results [20-22]. There seems to be a gap between the development of technology and usefulness in practice within health care organizations that needs to be bridged if technologies are to support health care rather than further increase its complexity [19].

### Addressing Complexity in Health Care Technology Projects

Greenhalgh et al [23] argued that adoption, scale-up, and spread of new technologies often fail due to complexity. They employed theories on complex adaptive systems and on the diffusion of innovations in health care to create a framework for using principles or rules to facilitate the development and application of technological innovations in complex contexts. The Nonadoption, Abandonment, and Challenges to Scale-Up, Spread, and Sustainability (NASSS) framework and complexity assessment tool (NASSS-CAT) were developed to help stakeholders identify and manage complexity in technology innovation projects in health care [8,23].

A university hospital in west Sweden started to develop a point-of-care dashboard to support patients and health care professionals in schizophrenia care. Successful prototyping and pilot testing led to the decision to scale-up the initiative to all the department's outpatient units. Challenges started to accrue when planning for larger-scale development and deployment of the dashboard and related tools. This case study evaluates the stakeholders’ use of the NASSS-CAT to inform the development and deployment of the dashboard and reports on the complexity profile of the project, stakeholders’ experiences when using a pilot version of the NASSS-CAT, and practical implications. This study also aims to inform a future multinational study with multiple cases having maximum intercase variation to field-test the NASSS-CAT [24].

## Methods

### Overall Design

This case study [25,26], informed by the principles of action research [27] and action evaluation [28], involved stakeholders’ use of the NASSS-CAT to inform the development and deployment of the point-of-care dashboard for patients and health care professionals in schizophrenia care. Another study will specifically evaluate patients’ experiences while using the dashboard at the point of care.

### Nonadoption, Abandonment, and Challenges to Scale-up, Spread, and Sustainability (NASSS) Framework

**Figure 1:**
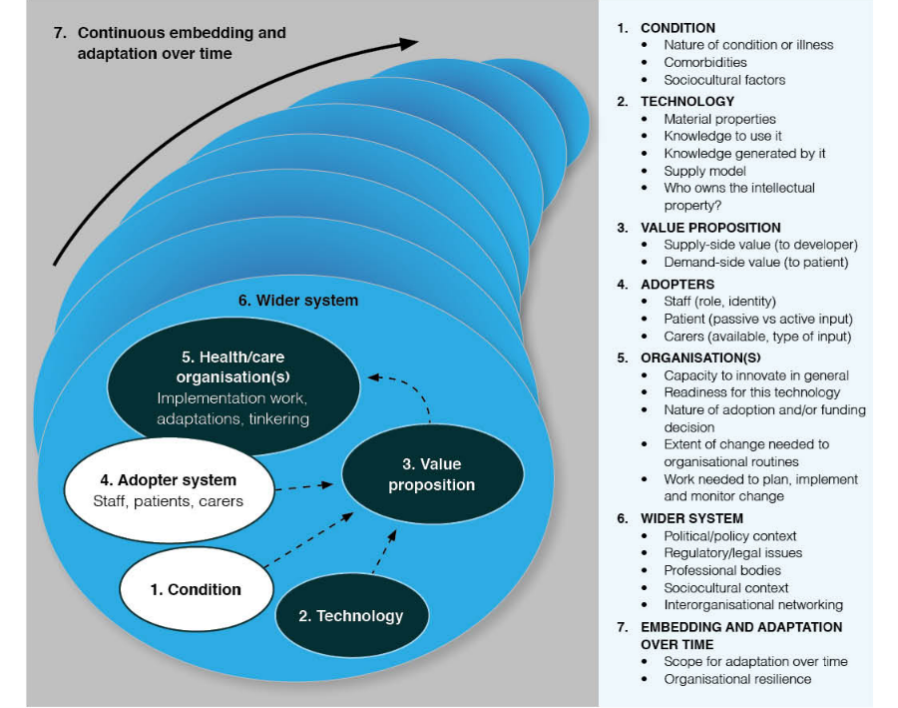
The Nonadoption, Abandonment, and Challenges to Scale-Up, Spread, and Sustainability (NASSS) framework by Greenhalgh et al [8]. Used with permission.

The developers of NASSS noted that it was crucial to understand the sociotechnical interaction between individuals, organizations, technology, and policy to explain why a new technology is adopted and sustained (or not) in health and social care [23,29]. The NASSS framework features 7 key domains, identified through systematic hermeneutic literature review and refined through empirical case studies of technology implementation [8]. It is intended to be used to guide and evaluate the success of technology deployment in sociotechnical systems. By addressing questions in the tool’s domains, properties of the technology and adopting system are placed along a continuum ranging from simple to complicated to complex (Figure 1) [8]. Knowledge of a technology project’s domain-specific complexity can aid stakeholders to respond adaptively, lessen complexity, and strengthen their capability to handle complexity [30]. Principles or “simple rules” can act as recommendations to guide further development (Textbox 1) [30].

**Ten simple rules for managing complexity [30].**

1. Strengthen program leadership, which may be distributed across the project and across contributing disciplines.

2. Codevelop an overall vision for the project and maintain dialogue around that evolving vision.

3. Nurture key relationships between individuals and organizations.

4. Develop individuals and encourage them to solve local problems creatively.

5. Make resources available for creative individuals and teams to use for generating solutions to local challenges.

6. Capture data on progress and feed it into ongoing deliberations.

7. Acknowledge and address the concerns of frontline staff.

8. Work with intended users to codesign technologies and the work routines they are intended to support, building in adaptability.

9. Control scope creep.

10. Address regulatory and policy barriers.

### Nonadoption, Abandonment, and Challenges to Scale-up, Spread, and Sustainability Complexity Assessment Tool (NASSS-CAT)

The NASSS-CAT has two primary components. The initial component, based on the NASSS framework, supports the development of a rich narrative that surfaces key areas of uncertainty and interdependence in the project. The second component, based on an adapted version of the complexity assessment tool by Maylor et al [31], consists of a series of questions to support emergent project planning and evaluation and, in particular, to prompt project teams to consider how they might either reduce or manage complexity across the different NASSS domains.

### Case Project: Developing and Deploying New Technology

**Figure 2:**
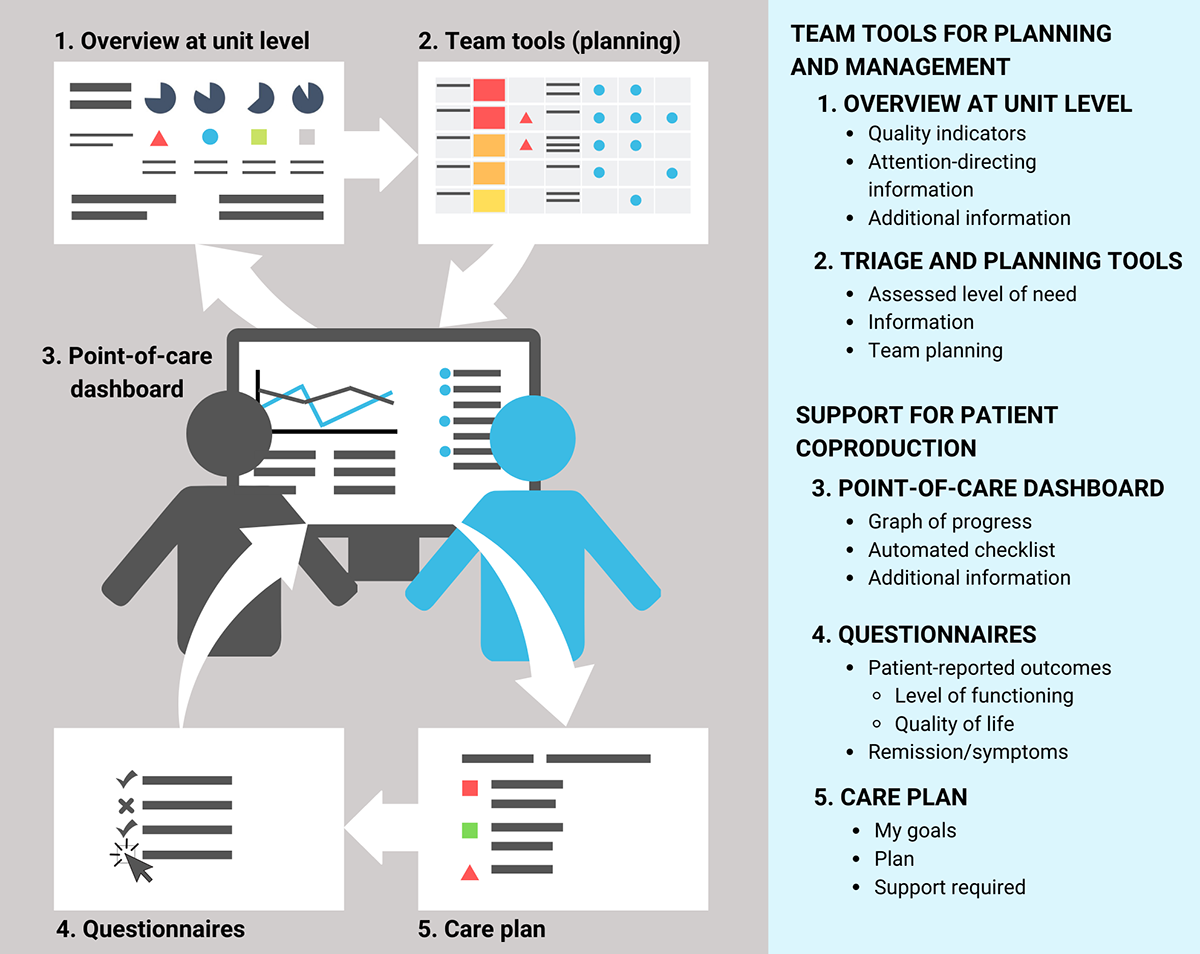
The dashboard and related tools: applications and visualizations.

The Department for Schizophrenia Spectrum Disorders at Sahlgrenska University Hospital in Gothenburg, Sweden (the Department) delivers specialized care for people with psychotic disorders in the metropolitan Gothenburg area (population of approximately 600,000 people). It serves 2600 patients, with schizophrenia as the most common diagnosis, at 8 outpatient units. About 20% of these patients need acute inpatient care at one of the Department’s 5 wards each year. To support patient engagement at the point of care, the Department developed a digital dashboard to visualize key indicators of each patient’s health and care status. The dashboard is one of several connected applications and displays to visualize data fed by several systems developed for several years and piloted at 2 outpatient units with some 400 patients (Figure 2). It includes team tools for care planning and management and tools to support coproduction of health and care among patients, their family members, and psychiatry staff. These tools include a unit-level overview of quality indicators identifying patients at risk, triage and planning tools including support for patient coproduction, a dashboard to be jointly reviewed at the point of care by patients and case managers/psychiatrists to support evaluation and planning, outcomes questionnaires, and patients’ care plans.

### Patient-perspective Vignette: The Role of the Technology

Below is a vignette from the fictitious patient Ana’s perspective [32] of a follow-up visit with her case manager at the outpatient clinic. It illustrates how the imagined dashboard-enhanced service model would differ from the traditional service model (pre-dashboard, Textbox 2).

Ana is a 34-year-old woman who has suffered from psychosis since her early twenties. After several diagnostic assessments, she was diagnosed with schizophrenia at age 28. Her medication treatment is combined with psychosocial interventions to help her manage her situation. On three occasions, psychotic episodes brought Ana into emergency inpatient care at the local hospital. She fears another episode and worries about how she would cope if it happened again. She is invited to annual check-ups as part of her continuous care at the outpatient unit.

**Predashboard situation compared with the imagined dashboard-enhanced service model.**

**Predashboard situation**

At her regular follow-up, it was hard for Ana to answer all the questions. Was she feeling better? Was there an increase in side effects? She struggled to remember how she felt the last time they jointly assessed her level of functioning and symptoms. She was not sure if the medication helped her. She dreaded making medication changes, for fear of new side effects. Both Ana and her case manager completed printed questionnaires. The content of the questionnaires provided some structure, covering important aspects of Ana’s situation. They were possibly useful for the clinic in documenting relevant information but did not help Ana in understanding her situation or lessening her anxiety.

**Dashboard-enhanced service model**

Using the point-of-care dashboard not only makes it easier to complete the questionnaires and review the care plan but also shows Ana’s progress and changes over time. An automated checklist signals to the case manager that it is time to update Ana’s care plan and perform a general health assessment within 3 months. Ana and her case manager review the digital visualization of Ana’s care plan and progress. Although Ana has not been feeling well over the past week, she is comforted by seeing how her symptoms and level of functioning have changed over time. Things are moving in the right direction. More than 2 years have passed since she had her last psychotic episode. Ana actively discusses the care plan with her case manager and psychiatrist, and they jointly update her goals.

### Collecting Case Study Data

A workshop using NASSS-CAT was set up for the project stakeholders. The focus of the workshop was to increase understanding of the influence of complexity in digitalization projects, determine the complexity profile of the dashboard project, explore the usefulness of the NASSS-CAT, and reflect on ways to manage complexity. The 11 participants were line managers, department directors, organization developers, and programmers representing different professions: psychiatrist, psychologist, occupational therapist, and IT developer. Before the workshop, participants received written and oral information about the study and the voluntary nature of their participation. All invited participants gave their informed consent and agreed to join. The day included both small breakout group sessions and large group discussions. Data collection was framed according to the NASSS-CAT and included observations, field notes, notes from the participants, and audio recordings of discussions during the workshop that were subsequently transcribed. The use of NASSS-CAT yielded a complexity profile and the second part of NASSS-CAT, with questions to prompt consideration of how to handle complexity, was particularly used to identify preliminary practical implications. The complexity profile and practical implications were later presented at two feedback sessions to department directors, managers, developers, and assistants involved in planning the future deployment of the dashboard project. Their discussion of the analysis served to validate the findings as a form of member checking [33] and to deepen understanding of practical implications in relation to the project. Data related to the use and usefulness of the NASSS-CAT from the workshop were analyzed by the authors (AG, ACA) using an inductive thematic approach inspired by Braun and Clarke [34]. The resulting themes and selected illustrative quotes are reported in the Results section in the subsection Experiences With Using NASSS-CAT. The piloting of the NASSS-CAT tools in health care settings within the United Kingdom had been approved by the UK Health Research Authority, Health Research Wales, and Health Research Scotland (IRAS no. 258679; REC no. 19/LO/0550). No formal ethical review was required for piloting the tools in Sweden.

## Results

The results are presented under three headings: complexity profile, experiences with using NASSS-CAT, and practical implications.

### Complexity Profile

Complexity mapping of the 7 NASSS-CAT domains showed significant complexity in 6 of the 7 domains. The domain of intended adopters (ie, health care professionals and patients at the point of care) was perceived to be the least complex.

#### The Condition or Illness

Schizophrenia is considered to be a complex condition due to its high level of multimorbidity [3,35] and the associated need for multimodal treatment and coordination of care between multiple providers of health and social care [5]. Despite extensive research on schizophrenia and the effectiveness of multimodal treatment programs, challenges remain regarding the successful coordination of multiple providers.

Due to cognitive impairment, persons with schizophrenia have varying degrees of insight into their condition and motivation, which affects adherence to treatment including medication, sometimes resulting in involuntary care and a need for coercive measures. Access to individualized treatment, housing, and support also varies substantially.

#### The Technology

The dashboard was developed within the Department in collaboration with other psychiatric departments at the hospital. It has significant technical interdependencies with systems controlled by the regional information technology (IT) department. The development of the dashboard has been intertwined with older systems, making use of work processes already in place. There are uncertainties on how to adapt the technology to enable scale-up across the whole department. To what extent the technology will be obsolete within 3-5 years is unknown, but the IT department plans the broad implementation of other new health information systems within that timeframe.

#### The Value Proposition

The value proposition of the project is uncertain. Case managers report finding the technology useful, as do patients, according to preliminary data. Local testing and piloting have generated evidence of perceived effectiveness, although the degree of cost effectiveness remains unknown. The staff spends less time on related administration. The dashboard provides an overview of patients’ progress and risks and supports collaborative planning of care.

The technology’s potential value as a commercial product is uncertain and probably impossible to assess because the new technology is interwoven with older systems.

Additional uncertainties are related to the IT department’s role in the maintenance and related costs.

#### The Intended Adopters

The domain of intended adopters is the least complex domain due to a perceived readiness within the organization. The primary users are health care professionals in the care team as well as patients during visits to the outpatient clinics. Secondary users include managers and administrators. The technology is expected to lessen the workload for administrators since more tasks are completed at the point of care by the patient and health care professional. The dashboard pilot testing at 2 outpatient units for 12 and 20 months, respectively, indicated that the innovation is useful for both health care professionals and managers. Furthermore, participating health care professionals report that most patients use the dashboard with ease at yearly follow-up visits. Most patients would prefer to have the next such visit include the dashboard.

#### The Organization(s)

The Department and technology have a good organization-innovation fit, as the innovation was developed in-house to support the organization’s mission and ambitions. Digitalization is perceived as a quality improvement strategy rather than as product development. Horizon scanning has increased the awareness of innovative technologies, and the organization has a tradition of supporting and trying new ways of working. In recent years, technological innovations have been a focus, enabled by recruitment of IT developers, and embedded in the organization. It has been challenging to pilot and evaluate new technical innovations due to dependencies on the regional IT department. The development of a dashboard is neither part of a regional initiative to develop the future health information system nor part of a product development plan with a clear business case. Internal support has made the pilot tests possible at the Department, but the lack of sponsors at higher organizational levels and uncertainty of the value proposition from a wider organizational perspective add challenges within this domain.

#### The Wider Context

Changes in the wider context may impact the organization and the introduction of the technology. In particular, implementation of a new health information system can potentially crowd out efforts to deploy the dashboard technology within the Department. There is an enormous drive for innovation and digitalization in Swedish health care, either in the form of large national or regional projects that are deemed hard to influence, or as small projects such as freestanding apps that cannot make use of available health care data. There are few opportunities to learn from other organizations; almost no other organization exists that uses similar technologies, and if they do, they mostly concern patient groups other than those within mental health services.

#### Project-Specific Complexity

The specific project to develop and deploy the technology across the Department brings challenges related to technical, structural, operational, and sociopolitical complexities. These include the fact that the technology does not yet exist in a robust and dependable form and that regulatory requirements related to secure authentication and access to patient-specific data are not finalized.

Structural and operational complexities include the fact that the technology depends on several other systems to access data. Lines of responsibility for tasks and deliverables are not yet defined, and there is a high dependency on key individuals in a small development team. The people managing the project are not wholly allocated to the project and do not have adequate control over resources, including project staff. Other key projects, particularly the new health information system implementation, can have a major impact on the project.

Sociopolitical complexities stem from the lack of a senior sponsor in the larger hospital and health system organization who recognizes the benefits of the dashboard initiative and can facilitate its progress. Its internal value proposition (within the Department) is clearer than a possible external business case, implying that organizational benefits, costs, and risks are largely unknown.

### Experiences With Using the Nonadoption, Abandonment, and challenges to Scale-Up, Spread, and Sustainability Complexity Assessment Tool (NASSS-CAT)

The thematic analysis of data for the stakeholders’ experiences of the use and usefulness of NASSS-CAT complexity mapping yielded 3 themes: new insights, threshold to start using NASSS-CAT, and inclusion of relevant stakeholders. These are presented with illustrative quotes from workshop participants. Quotes were translated from Swedish.

#### New Insights

Using the tool in a workshop increased awareness of the role of complexity. Participants highlighted the importance of identifying complexity and of possibilities to address it in this project in particular and in other future projects in general. Opinions varied about when in the process the tool would bring the greatest benefit or whether it ought to be used throughout the whole project.

(Complexity mapping) supports getting perspective and new insights that might help in addressing challenges differently.

Participant 2, director

I think this (the NASSS-domains) is helpful in (identifying) what to consider in the different digitalization projects and deployment initiatives before getting on with it.

Participant 5, developer

#### Threshold to Start Using the Nonadoption, Abandonment, and Challenges to Scale-Up, Spread, and Sustainability Complexity Assessment Tool (NASSS-CAT)

Participants stated that a basic understanding of the assessment tool´s core concepts would be helpful before starting to use it. During the workshop, the participating authors needed to interpret several NASSS-CAT concepts in light of the local dashboard context for workshop participants (eg, does the “organization(s)” include the IT department or do “users” also include any family members present at a patient’s visit to the outpatient unit?).

We discussed the meaning of different concepts and had different interpretations. Maybe preparations could have helped.

Participant 1, manager

Less time had been necessary to use to gain a mutual understanding of concepts, if they, in advance, had been defined more specifically in relation to the project.

Participant 4, manager

#### Inclusion of Relevant Stakeholders

Several challenges surfaced during the workshop that could not be addressed directly because they depended on functions or parts of the organization that were not represented at the workshop, such as the IT department. Participants wished to include them in the complexity assessment to get a better understanding and in the development and deployment of the technology to increase the chances of success. Another issue discussed during the workshop was the importance of creating shared experiences and insights for staff and leaders by allocating time to participate in such an exercise.

I think it could have been a very interesting discussion if other parts of the organization had been represented here with more people with other perspectives on these matters. It would have been great.

Participant 4, manager

I believe it would have been important for more people to attend today to share some of the experiences. It creates more power to move on, actually.

Participant 2, director

### Practical Implications

By using the second part of the NASSS-CAT in particular, with questions to prompt consideration of how to handle complexity, the simple rules for managing technology projects in complex systems (Textbox 1), and the feedback from directors and managers on the complexity analysis through the feedback and validation sessions, the following practical implications were identified, serving as project-specific recommendations:

1. Develop a clear value proposition with information on costs, benefits, and risks. This can guide decisions to make more resources available or to halt further development.
2. Update the overall vision and maintain dialogue to keep it common and up-to-date as the initiative evolves (Rule 2).
3. Strengthen the project’s leadership and support structure by clarifying how the project is governed and organized. Earmark resources for it in terms of both money and dedicated time of key individuals (Rules 1 and 5).
4. Maximize benefits and minimize complexity by focusing on parts of the technology/innovation with a low(er) threshold to deployment (Rules 4-9) related to the front-line users: case managers, psychiatrists, and patients. Set and keep the scope of the development and deployment project by using measures to monitor and understand progress and benefits such as saving time, reducing administration, and gaining a better overview.
5. Act strategically in the wider context (Rules 9 and 10) to strengthen the initiative by creating a strategy and plan for communication upwards in the organizational hierarchy and outwards to gain acceptance and sponsorship from key individuals (Rule 3), considering if “rebranding” can make the dashboard’s development and its work processes better fit into the policy context, and connecting to other departments that develop, use, or evaluate similar technologies.

## Discussion

### Principal Findings

The dashboard initiative’s complexity profile – with considerable complexity demonstrated in 6 of 7 domains – indicates that the initiative is unlikely to proceed successfully under current circumstances, reflecting the observation of Maylor et al [31]: “the greater the complexity posed by a project, the lower the chance that any successful outcome, let alone an innovative one, will be achieved.” Nevertheless, using complexity as a lens when assessing the initiative was perceived as meaningful since it revealed not only challenges but also strengths. Developing the dashboard locally resulted in a high level of engagement and readiness to participate among both health care professionals and patients in focus groups, user testing, and pilots. The collaboration and codesign of technologies and work routines in the local development project might explain why participating stakeholders in the workshop perceived the domain of “intended adopters” to be the least complex when considering further deployment of the dashboard. Perhaps the greatest remaining complexity in the dashboard project concerns the challenges of connecting top-down and bottom-up initiatives and processes, whether related to the development and spread of the dashboard or the related technology, goal alignment, or governance.

### Methodological Considerations

There are several limitations to this study. It is a small, single case study of an application of the NASSS-CAT to see if it could be useful in a local development and deployment project. It is restricted to use in the local project and does not test the usability and usefulness of the tool at higher levels in the organization, such as macro-level leadership or involvement from the regional IT department, even though the project has strong interdependencies to those parts of the organization. Patient involvement in the use of the complexity assessment tool could have helped gather further useful information, but the involvement of patients has been restricted to the development of the digital dashboard and not the use of NASSS-CAT. Assessing the complexity of a technology project can aid in understanding and planning but may not be enough on its own to identify what needs to be addressed to succeed with scale-up and spread of innovations [36]. Further research is needed to identify how complexity assessment, using the NASSS-CAT, at various levels of organizations from individuals to the top management can support the development and deployment of new technologies in complex health care contexts.

### Conclusions

Complexity assessment of the dashboard project using the NASSS-CAT helped highlight important areas and challenges identified through rigorous research as important in the development and deployment projects of new technologies in health care settings. Experiences from the workshop and validation sessions showed that domains that otherwise might have been neglected received more attention and were brought forward to subsequent planning of the project. The assessment identified strengths of the dashboard initiative to further build upon, while also exposing wider organizational complexity that can challenge the process and spread of the initiative. The assessment helped stakeholders generate specific ideas for how to reduce complexity and strengthen the ability to manage any remaining complexity. This pilot testing of the NASSS-CAT in a real-life setting suggested that the NASSS-CAT can provide participants with a greater understanding of complexities in digitalization projects in general.

## Abbreviations

IT: information technology.
NASSS: The Nonadoption, Abandonment, and Challenges to Scale-Up, Spread, and Sustainability framework.
NASSS-CAT: The Nonadoption, Abandonment, and Challenges to Scale-Up, Spread, and Sustainability framework and complexity assessment tool.
